# Characterization of Biosensors Based on Recombinant Glutamate Oxidase: Comparison of Crosslinking Agents in Terms of Enzyme Loading and Efficiency Parameters

**DOI:** 10.3390/s16101565

**Published:** 2016-09-23

**Authors:** Rochelle Ford, Susan J. Quinn, Robert D. O’Neill

**Affiliations:** UCD School of Chemistry, University College Dublin, Belfield, Dublin D04 N2E5, Ireland; Rochelle.Ford@ucdconnect.ie (R.F.); Susan.Quinn@ucd.ie (S.J.Q.)

**Keywords:** glutamate biosensor stability, biomedical applications, amperometry, surface enzyme loading and affinity, permselective polymer, poly(*ortho*-phenylenediamine)

## Abstract

Amperometric l-glutamate (Glu) biosensors, based on both wild-type and a recombinant form of l-glutamate oxidase (GluOx), were designed and characterized in terms of enzyme-kinetic, sensitivity and stability parameters in attempts to fabricate a real-time Glu monitoring device suitable for future long-term detection of this amino acid in biological and other complex media. A comparison of the enzyme from these two sources showed that they were similar in terms of biosensor performance. Optimization of the loading of the polycationic stabilization agent, polyethyleneimine (PEI), was established before investigating a range of crosslinking agents under different conditions: glutaraldehyde (GA), polyethylene glycol (PEG), and polyethylene glycol diglycidyl ether (PEGDE). Whereas PEI-free biosensor designs lost most of their meager Glu sensitivity after one or two days, configurations with a 2:5 ratio of dip-evaporation applications of PEI(1%):GluOx(400 U/mL) displayed a 20-fold increase in their initial sensitivity, and a decay half-life extended to 10 days. All the crosslinkers studied had no effect on initial Glu sensitivity, but enhanced biosensor stability, provided the crosslinking procedure was carried out under well-defined conditions. The resulting biosensor design based on the recombinant enzyme deposited on a permselective layer of poly-(*ortho*-phenylenediamine), PoPD/PEI_2_/GluOx_5_/PEGDE, displayed good sensitivity (LOD < 0.2 μM), response time (*t*_90%_ < 1 s) and stability over a 90-day period, making it an attractive candidate for future long-term monitoring of Glu concentration dynamics in complex media.

## 1. Introduction

Research into the design and characterization of biosensors for l-glutamate (Glu) is currently a significant area of study due to the important roles this amino acid plays in the food industry [1,2,3] and as a neurotransmitter [4,5]. In the latter context, Glu is the most widespread excitatory neurotransmitter in the mammalian brain, and has been implicated in a number of psychiatric and neurological disorders, such as schizophrenia, Parkinson’s disease, and stroke [6,7,8,9,10]. However, there are many challenges associated with exploring the roles of brain Glu, using different *in vivo* monitoring techniques. For example, high sensitivity is required because of the low baseline concentration of Glu in extracellular fluids (ECF) of the intact living brain (i.e., *in vivo*), estimated in the range of 1–10 µM which can depend on a wide variety of conditions, such as the anatomical location of the implanted probe and the level of anaesthesia [8,9,11,12,13]. A wider range of brain Glu concentration values have been reported under more extreme conditions. For example, for slices of excised brain (i.e., *in vitro*) levels as low as 25 nM have been estimated [14]. In contrast, values of hundreds of micromolar have been detected *in vivo* when baseline levels of ~2 μM were increased by two orders of magnitude following traumatic brain injury [15].

Both invasive and non-invasive technologies have been developed for neurochemical monitoring *in vivo*. While the latter have many attractions associated with the absence of tissue disruption, and some are sensitive enough to detect behavior-correlated neurotransmitter release [16], they are currently not suitable for chronic studies. The two main approaches to measuring long-term brain Glu levels *in vivo* are microdialysis [17,18] and electrochemical biosensors [13,19,20]. A temporal resolution of ~1 min has been achieved using microdialysis for the detection of neurotransmitters such as serotonin [21] and Glu [22]. In contrast, biosensors represent a more useful approach for monitoring fast concentration dynamics of Glu (sub-second scale) due to the higher spatial and temporal resolution achievable with implantable amperometric biosensors. Moreover, long-term *in vivo* electrochemistry (LIVE) has been demonstrated in the case of sensors, such as carbon-fiber or carbon-paste electrodes for monitoring neurotransmitters [23] and their metabolites [24] over periods of months. However, the inherent complexity of biosensors, incorporating a range of components (including enzymes, permselective membranes and enzyme stabilizers), aggravates the challenge of their long-term stability in biological tissues and other complex media [24,25,26]. In this work, a biosensor was designed for the detection of Glu, with an emphasis on sensitivity and stability, based on both wild-type and recombinant l-glutamate oxidase (GluOx) as the sensing element.

The enzyme reactions of GluOx to produce electroactive hydrogen peroxide (HP) can be written as Equations (1)–(2).

l-Glutamate + H_2_O + GluOx/FAD → α-ketoglutarate + NH_3_ + GluOx/FADH_2_ (1)

GluOx/FADH_2_ + O_2_ → GluOx/FAD + H_2_O_2_ (2)

H_2_O_2_ → O_2_ + 2H^+^ + 2e^−^ (3)

Oxidizing this enzyme-generated HP (Equation (3)) requires a relatively high applied potential (0.4–0.7 V vs. SCE [27,28,29]) to provide good HP sensitivity. At these potentials, however, endogenous reducing agents, such as ascorbic acid (AA), which is present in high concentrations in most biological tissues and fluids, can give rise to false positive biosensor responses *in vivo*. A permselective barrier is therefore essential in the design of implantable biosensors, and a range of electro-deposited poly-phenylenediamines [30,31,32,33,34,35] and polyphenols [36,37,38,39] have been developed to improve biosensor selectivity for brain monitoring where ECF ascorbate levels are particularly high (up to 1 mM) [40,41].

Although polyphenols show exceptional AA-blocking characteristics [38], polyphenylenediamines are commonly the permselective membrane of choice for biosensor applications because of their high permeability to HP [31,42,43,44,45], in addition to excellent AA rejection. For analytical environments containing levels of AA greater than 0.2 mM, it has been suggested that poly(*o*-phenylenediamine), PoPD, is superior to poly(*m*-phenylenediamine) in terms of biosensor permselectivity [44,46], and was, therefore, the interference-rejection polymer of choice in this work.

Here, we build on previous studies of PoPD-modified, wild-type GluOx-based biosensors designed for LIVE applications which have demonstrated, in addition to outstanding permselectivity, the following features: electrostatic stabilization [47] of GluOx with polycationic polyethyleneimine, PEI [26,48,49]; low limits of detection (<1 µM) [50,51]; fast response times (~1 s) [51]; and their negligible oxygen dependence (Equation (2)) for concentrations of Glu and O_2_ relevant to neurochemical monitoring [48,50]. However, their long-term stability was limited (days) [52]. A key aim here, therefore, was to fabricate biosensors which would be suitable for chronic implantation in brain ECF, using biosensor optimization parameters (enzyme loading, affinity, and analytical sensitivity [20]), as well as stability parameters, as guidelines.

Initial work involved comparing a more readily available recombinant GluOx [53] with the wild-type enzyme [54] used previously in a basic PoPD/PEI/GluOx biosensor design. Then, having fine tuned the PEI/GluOx ratio, the additional strategy for engendering good stability, coupled with high values of enzyme loading, substrate affinity and sensitivity, was to include a crosslinking agent capable, inter alia, of reacting with functional groups of the three main components in the polymer-enzyme composite (PEC) layer. These crosslinkers included glutaraldehyde (GA), polyethylene glycol (PEG), and polyethylene glycol diglycidyl ether (PEGDE) introduced under different conditions. PEGDE has been used in the past [55] and more recently as a more favourable crosslinker compared with GA [56,57] for the immobilization of GluOx to produce higher and more stable sensitivities. It was hoped that PEGDE would be a more suitable modifier here as it is a less disruptive crosslinker than GA, retaining greater GluOx catalytic activity [57]. Recent advances in the area of Glu biosensor development include the use of cryopreservation to retain high sensitivity to Glu for 30 days [56]. Shelf-life has also been examined, with some biosensor designs being able to retain their sensitivity to Glu after implantation [58] and for up to four months of storage [59].

By incorporating PoPD, PEI and a crosslinking agent into the biosensor design it was hoped that it would be suitable for future LIVE monitoring of Glu. Moreover, the biosensor fabrication protocols used here (electro-deposition and dip coating) lend themselves to further miniaturization, say to a micro-electrode-array (MEA) format (40–50 µm diameter) that does not induce as much tissue damage as larger probes [60], or other complications associated with damage to brain tissues [61,62,63].

## 2. Materials and Methods

### 2.1. Reagents and Solutions

A 200 U·mL^−1^ solution of native, and a 400 U·mL^−1^ solution of recombinant, glutamate oxidase (GluOx, *Streptomyces* sp. X-119-6; EC 1.4.3.11) in phosphate buffered saline (PBS) solution were supplied by Enzyme-Sensor Co. Ltd. (Tsukuba, Japan), and stored at −21 °C when not in use. The 400 U·mL^−1^ solution of recombinant enzyme (expressed in *Escherichia coli* and proteolyzed with an metalloendopeptidase from *Streptomyces griseus* [53]) was used here throughout, unless otherwise stated. Glutamate (Glu), *o*-phenylenediamine (oPD), polyethyleneimine (PEI, 50% *w*/*v* aqueous solution, ~750 kDa), HCl (1 M), H_2_O_2_ (3%, *w*/*w*), PBS tablets, glutaraldehyde (GA, 25% *w*/*v*), polyethylene glycol (PEG) and polyethylene glycol diglycidyl ether (PEGDE) were all obtained from Sigma, and used as supplied. PBS stock solutions (pH 7.4) were prepared in Milli-Q^®^ water (18.2 MΩ·cm), and stored at 4 °C as were the stock analyte solutions. A 1% PEI solution was prepared by diluting the received PEI solution in H_2_O. A 300 mM monomer solution of oPD was prepared in 10 mM HCl. A 0.3% *w*/*v* solution of H_2_O_2_ was prepared in deionized water and stored in the fridge. A 1% *w*/*v* GA solution was prepared in Milli-Q^®^ water and stored in an active fume hood.

### 2.2. Instrumentation and Software

Chart™ (ver. 5.2; AD Instruments Ltd., Oxford, UK) software for Windows was used for constant potential amperometry at an applied potential of either +500 mV (for calibrations) or +700 mV (for electropolymerizations) vs. a Ag pseudo-reference electrode (see Section 2.3). The Chart software was required to operate the Powerlab 8/30 (AD Instruments Ltd.) interfaced to the ACM-IV potentiostat (Biostat IV, ACM Instruments, Cumbria, UK). Prism (ver. 5.01; GraphPad Software, San Diego, CA, USA) was used for data analysis.

### 2.3. Amperometric Experiments

All experiments were done in a 20 mL glass cell containing PBS (pH 7.4) at an ambient 21 ± 1 °C, using a standard three-electrode set-up, including a silver pseudo-reference electrode (250 µm diameter Ag wire) whose potential in PBS was within 30 mV of a SCE (similar to that reported for Ag pseudo-references in other background electrolyte solutions with like chloride-ion concentration [27]), and a stainless-steel needle as the auxiliary electrode (0.8 mm diameter). The working electrodes were platinum-iridium wires (90:10 ratio, Advent Research Materials Ltd., Oxford, UK) stress-relieved Teflon^®^-insulated wire, 125-µm internal diameter. Glu and HP calibrations were performed in quiescent PBS (following stabilization of the background current for 1 h) by adding aliquots of Glu or HP stock solution to a stirred solution of PBS in the electrochemical cell, and allowing the solution to become quiescent. For electropolymerization in the 300 mM oPD solution, an optimized potential of +0.7 V vs. the Ag pseudo-reference electrode was applied for 15 min [64].

### 2.4. Electrode Preparation and Modification

#### 2.4.1. Electrode Preparation

Approximately 2 mm of Teflon^®^ was removed from the Pt-Ir wire electrode using a scalpel in a rolling movement. This was cut more accurately using a monocle under a light microscope to 1.0 ± 0.1 mm. Approximately 2 mm of Teflon was removed from the other end of the wire and this was soldered to a gold clip to facilitate the connection of the working electrode to the potentiostat circuit. Compared with pure Pt, the mechanically more robust Pt-Ir working electrodes have similar electrochemical properties and are more generally used for implantable biosensors [44]; they are normally designated as Pt, however, for simplicity of biosensor notation (see Section 2.5).

#### 2.4.2. Electrode Modification

Dip evaporation was used to immobilize PEI and GluOx by quickly dipping the wire electrode into an Eppendorf tube containing the corresponding solution for ~1 s and then allowed to dry for 5 min at room temperature. This process was repeated a specified number of times to increase the amount of macromolecular material deposited. In some biosensor designs the enzyme was trapped on the surface of the biosensor, using either GA, PEG or PEGDE, by dipping the biosensor once into a solution containing the crosslinker at various concentrations and then cured under different conditions. For PEG and PEGDE crosslinking, the electrodes were dipped into a solution of the crosslinker for 1 s, and allowed to cure at room temperature for 10 min, unless stated otherwise. The electrodes were then rinsed in water for 5 min, immersed in a fresh solution of PBS in the electrochemical cell, a potential of +0.5 V applied, and allowed to settle for 1 h before calibration.

### 2.5. Biosensor Nomenclature

The order of deposition of the various biosensor components is important in optimizing biosensor functionality (sensitivity, selectivity and stability), and the nomenclature used to represent each design should reflect this order. In the system used here [20], Pt_C_ represents the Pt-Ir wire with a cylinder geometry, and the modifier deposition sequence is the same as in the nomenclature. For example, Pt_C_/PoPD/GluOx indicates that the PoPD polymer was electropolymerized directly onto the metal surface, followed by deposition of the GluOx enzyme. Dip evaporation is a technique commonly used to apply modifier to electrode surfaces [56,65], and varying the number of dips can be used facilely to build up the amount of modifier deposited. There was a need, therefore, to represent this with a number after the modifier. For example, Pt_C_/PoPD/PEI_2_/GluOx_5_/PEGDE (0.1%) represents a PoPD polymer-coated Pt electrode modified by two dips of PEI solution (a fixed concentration of 1% PEI was used throughout this study) followed by five dips of GluOx (fixed concentration of 400 U·mL^−1^ (as supplied) throughout this study), followed by crosslinking with 1 dip of 0.1% PEGDE.

### 2.6. Data Analysis

#### 2.6.1. Hydrogen Peroxide Sensitivity Ratio, HP%

Because HP is the enzyme signal-transduction molecule for the oxidase-based biosensors studied here (Equations (2) and (3)), we quantified the effect of various biosensor modifications on its HP sensitivity by measuring the HP response of the Pt before and after modification, HP(bare) and HP(bios), respectively, for each individual sensor. The HP calibration slope was linear in the concentration range studied (0–100 μM) for both bare Pt_C_ and PoPD-based polymer-enzyme composite biosensors, as observed previously [31,44]. The HP sensitivity was therefore quantified as the slope of the linear HP calibration plots, and their ratio (HP%) used to quantify the effect of the modification layers on the HP sensitivity; see Equation (4).

(4) $$\mathrm{HP}\%=\frac{\mathrm{HP}\left(\mathrm{bios}\right)\;\times \;100}{\mathrm{HP}\left(\mathrm{bare}\right)}$$

#### 2.6.2. Michaelis-Menten Parameters

**Figure 1:**
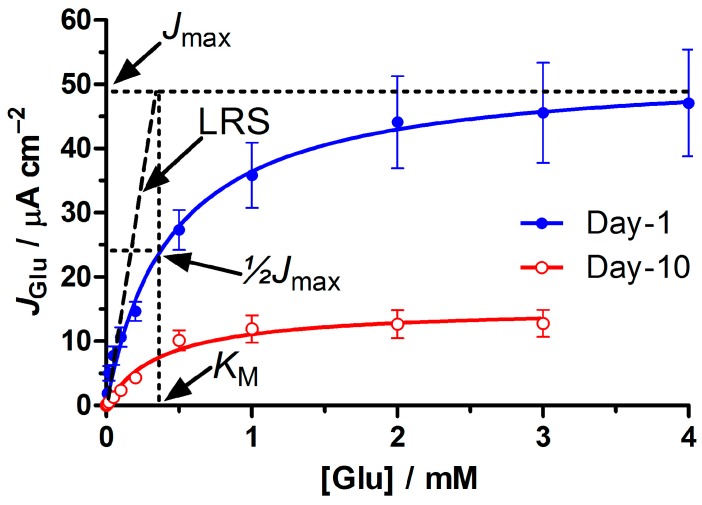
Initial studies using recombinant GluOx in combination with PEI, but without a permselective barrier or crosslinking agent. Calibration data and non-linear regression (Equation (5); *R*^2^ = 0.985 ± 0.003) for the Pt_C_/PEI_2_/GluOx_5_ design illustrating the Michaelis–Menten kinetic parameters, *J*_max_ and *K*_M_, shortly after fabrication, as well as loss of active surface enzyme by day 10.

Oxidase-based biosensor responses are often essentially hyperbolic, and can be modeled using a Michaelis-Menten kinetic analysis (see Figure 1) [44]. Equation (5) is the enzyme-kinetic equation expressed in terms of current density (*J*_S_) of the background-corrected biosensor response to substrate (S) at a concentration [S]. *J*_MAX_ is the current that would be observed at enzyme saturation and *K*_M_ is the apparent Michaelis constant, an affinity parameter that defines the concentration of substrate, which gives half the *J*_MAX_ response (Figure 1). For each biosensor design, the current density values of the calibration were plotted against the Glu concentration and non-linear regression used to determine the *J*_MAX_ and *K*_M_, using Equation (5). Although the biosensor response was hyperbolic over a wide range of concentrations (average Hill coefficient was 1.3 ± 0.3 (SD), *n* = 30, and not very different from unity), it is approximately linear up to ~½ *K*_M_, with a corresponding linear region slope (LRS) value which is often used to quantify biosensor sensitivity to substrate. The limit of the LRS as [S] → 0 is the maximum slope, with a value of *J*_MAX_/K_M_ [20]; see Figure 1. The average coefficient of determination (*R*^2^ value) for the LRS from a large sample population was 0.990 ± 0.002, *n* = 100. Therefore, both Michaelis–Menten non-linear and linear regression analyses were used for Glu calibrations. Linear regression analysis only was needed for HP calibrations.

(5) $$J_{S\;}=\;\frac{J_{\mathrm{MAX}}}{1+\;K_{M}/\left[S\right]}$$

#### 2.6.3. Normalized Enzyme Parameters, BE% and Enz_act_

Because the biosensor response to substrate Glu depends on its HP sensitivity (Equations (1)–(3)), a number of key parameters such as *J*_MAX_ and LRS (see Figure 1) were normalized with respect to biosensor HP sensitivity. The biosensor efficiency or BE% (Equation (6)) can be thought of as a measure of the efficiency of the biosensor in converting substrate (S) to HP [20]. This parameter was used because there may be some variation in the Glu LRS caused by changes in the sensitivity of the biosensor to HP, such as electrode aging; the LRS was therefore normalized with respect to the biosensor HP slope, and since the LRS has a limiting value of *J*_MAX_/*K*_M_ [20], BE% reflects the major enzyme parameters determining biosensor response to substrate (loading of active enzyme and enzyme affinity), but is independent of HP sensitivity (Equation (6))*.* The maximum observed value of BE% to date is of the order of 60% [44,47], which corresponds to Glu being converted to HP at a diffusion-limited rate, coupled with a significant fraction of enzyme-generated HP molecules not being oxidized at the electrode because of loss to the bulk solution [66].

(6) $$\mathrm{BE}\%=\;\frac{\mathrm{LRS}\;\times 100}{\mathrm{HP}\left(\mathrm{bios}\right)}=\frac{J_{\mathrm{MAX}}\times 100}{K_{M}\times \mathrm{HP}\left(\mathrm{bios}\right)}=\frac{{\mathrm{Enz}}_{\mathrm{act}}\times 100}{K_{M}}$$

Enz_act_ (Equation (7)) has been used in the past to give an indication of active enzyme loading on the biosensor [20], and is *J*_MAX_ normalized with respect to the HP slope. Enz_act_ can give a more accurate indication of active enzyme loading than *J*_MAX_ because variations in *J*_MAX_ may occur which are due to changes in biosensor HP sensitivity, enzyme loading or enzyme activity. Thus, by normalizing *J*_MAX_ with respect to HP sensitivity it is possible to compare the loading of *active* enzyme molecules that contribute to the biosensor response across different biosensor designs. For example, enzyme molecules that are located far from the Pt electrode surface (say, stacked on multiple layers of PEI) might not contribute significantly to the biosensor response because most of the enzyme-generated HP may diffuse to the bulk solution before it can be electro-oxidized. The units of Enz_act_ are given in mM, and represent the concentration of hydrogen peroxide that gives the same biosensor response as the Glu *J*_max_ value.

(7) $${\mathrm{Enz}}_{\mathrm{act}}=\frac{J_{\mathrm{MAX}}}{\mathrm{HP}\left(\mathrm{bios}\right)}$$

### 2.7. Statistical Analysis

All data are reported as mean ± standard error (SEM), with *n* the number of electrodes, unless stated otherwise. Student’s *t*-tests were used to compare different biosensor configurations, as well as time-dependent changes for a given design, and the difference deemed to be statistically significant for *p* < 0.05, using a 95% confidence interval. A common estimate of limit of detection (LOD) [34] was calculated for these biosensors, using Equation (8), where SD is the standard deviation of the baseline biosensor current in background electrolyte.

LOD = 3.3 SD/LRS
(8)

## 3. Results and Discussion

### 3.1. Optimizing the Ratio of PEI to GluOx

PEI, a polycationic polymer, is commonly used for the immobilization and stabilization of enzymes on biosensor surfaces [47,67,68,69], and was used here in all biosensor designs to help neutralize the electrostatic charge associated with high loading of anionic GluOx on the electrode surface. This is particularly important when the enzyme substrate is anionic, as in the case of Glu detection at pH values close to neutrality [47]. In the absence of PEI, a high surface loading of wild-type GluOx has been shown to result in a high *K*_M_ value, i.e., a low affinity of Glu for the enzyme, which was reversed by surface PEI [26,48]. The balance of PEI and recombinant GluOx loading was therefore investigated here to provide the best analytical parameters (especially sensitivity and stability) for long-term Glu monitoring. Varying amounts of PEI and GluOx (PEI/GluOx_2_, PEI/GluOx_5_, PEI_2_/GluOx_2_, PEI_2_/GluOx_5_, PEI_5_/GluOx_5_, and PEI_4_/GluOx_10_; see Section 2.5 for biosensor nomenclature) were dip evaporated onto the bare Pt wire cylinder electrodes, and calibrations performed to compare levels of enzyme loading, substrate affinity, and biosensor efficiency (see Equations (5)–(7)). These initial studies focused on relatively freshly-made biosensors (calibrated daily up to day 10). Quantification of long-term biosensor stability (up to 90 days) was carried out later on the fine-tuned designs.

Glutamate calibrations of all Pt_C_/PEI_m_/GluOx_n_ designs followed Michaelis-Menten kinetics (see Figure 1). For these initial studies using recombinant GluOx in combination with PEI, but without a permselective barrier or crosslinking agent, the Glu sensitivity tended to decrease on the days following biosensor fabrication (see Figure 1). This sensitivity can be expressed either in terms of the biosensor response when the enzyme is saturated (*J*_max_) or the slope of the linear region (LRS = *J*_max_/*K*_M_ [50]; see Figure 1). The former is a useful index of the loading of active enzyme on the surface, but is also affected by biosensor hydrogen peroxide (HP) sensitivity (Equations (1)–(3)). Here, therefore, each *J*_max_ value was normalized with respect to the HP sensitivity determined by HP calibration of the corresponding biosensor. Using Equation (7), this provides Enz_act_, a parameter which more closely reflects variations in the loading of active enzyme across a range of biosensor configurations, or during the aging of a specific design (see Section 2.6.3 for definitions and the significance of the units).

#### 3.1.1. GluOx Loading Parameter, Enz_act_

**Figure 2:**
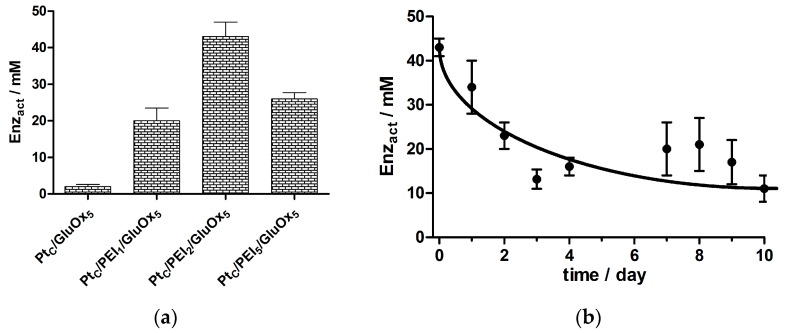
Initial studies using recombinant GluOx in combination with PEI, but without a permselective barrier or crosslinking agent: (**a**) Comparison of day-0 HP-normalized *J*_max_ value (Enz_act_; see Equation (7)) for Pt_C_/PEI_X_/GluOx_5_ designs, with x = 0, 1, 2 and 5 (see text for discussion); (**b**) Time course of data and trend-curve up to day 10 for the Pt_C_/PEI_2_/GluOx_5_ design showing loss of active enzyme loading, using the Enz_act_ values.

The importance of surface PEI in biosensors designed for long-term monitoring of Glu was demonstrated by the behavior of the PEI-free configuration (Pt_C_/GluOx_5_), which displayed a low normalized *J*_max_ value (Enz_act_, 2.0 ± 0.3 mM, *n* = 4) even for calibrations carried out on the day of fabrication (day 0; see Figure 2a). Furthermore, this meagre loading level was lost completely by day 2: 0.01 ± 0.01 mM, *n* = 4.

The ratio of PEI to GluOx which gave the highest initial (day 0) Enz_act_ value was Pt_C_/PEI_2_/GluOx_5_ (43 ± 2 mM, *n* = 4; see Figure 2), resulting from a combination of high *J*_max_ and good biosensor sensitivity to hydrogen peroxide. A 40% lower Enz_act_ value was observed for the second-best biosensor configuration (Pt_C_/PEI_5_/GluOx_5_, 26 ± 1 mM, *n* = 3; *p* < 0.005 compared with the Pt_C_/PEI_2_/GluOx_5_ design). The lowest Enz_act_ value was seen for the Pt_C_/PEI/GluOx_2_ design (19 ± 2 mM, *n* = 3), which was significantly lower than for the Pt_C_/PEI_2_/GluOx_5_ design (*p* < 0.001). These day-0 results highlight the importance of the balance of surface PEI and GluOx, rather than simply the amount of each compound deposited, in order to achieve good initial enzyme loading.

Finally in this context, a doubling of the number of dips for the best PEI:GluOx ratio (2:5), corresponding to a Pt_C_/PEI_4_/GluOx_10_ design, gave a poorer Enz_act_ value on day 0 (26 ± 2 mM, *n* = 9) compared with the Pt_C_/PEI_2_/GluOx_5_ configuration (*p* < 0.001), and more closely resembled the active enzyme loading of Pt_C_/PEI_5_/GluOx_5_ (*p* > 0.95). These results are consistent with the interpretation of Enz_act_ given in Section 2.6.2: excessive layers of macromolecular PEI (~750 kDa) increase the average distance between GluOx and the Pt surface, leading to increased diffusional loss of enzyme-generated HP to the bulk, and a corresponding lowering of biosensor response. The finding that the Pt_C_/PEI_2_/GluOx_5_ design was superior to the other configurations of PEI and recombinant GluOx explored here is consistent with the same conclusion drawn from a comparison of biosensors based on wild-type GluOx [47]; see also Section 3.3.

There was no clear trend in the effect on HP sensitivity (linear calibration slope, μA·cm^−2^·mM^−1^) for different loading levels of PEI and GluOx on the Pt surface, and the data were therefore pooled (*n* = 16): bare Pt_C_ (179 ± 20) and Pt_C_/PEI_m_/GluOx_n_ (138 ± 23). Equation (4) gave a HP% value of 77% ± 15% (*p* = 0.06 compared with the ideal value of 100%), indicating that the deposition of these macromolecules caused only a marginal decrease in biosensor HP sensitivity relative to bare Pt. 

There was also little variation in the *K*_M_ values across all the basic Pt_C_/PEI_n_/GluOx_m_ designs and, in contrast to the progressive loss of active enzyme molecules over the first 10 days of repeated calibration (see Figure 1 and Figure 2b), there was no statistically significant change in *K*_M_ over this period. The values were, therefore, pooled and expressed as a mean ± SD: 0.4 ± 0.1 mM (*n* = 104 determinations). This value for the surface-bound enzyme is expectedly greater than that reported for solution GluOx (0.23 mM for both the wild type [54] and recombinant type [53] used here; see explicit comparison in Section 3.3) because of restriction of Glu access to the biosensor GluOx by the surface itself, reducing the binding rate constant. In contrast, the increase in *K*_M_ caused by enzyme–substrate electrostatic repulsion in PEI-free Glu biosensor designs in a previous study was of the order of 1.5 mM for the upper loading levels seen here for biosensors incorporating PEI [50]. The maintenance of the mean *K*_M_ value below 0.5 mM for the Pt_C_/PEI_n_/GluOx_m_ designs indicates that the PEI was efficiently moderating the electrostatic repulsion between anionic surface enzyme and substrate [47].

#### 3.1.2. Biosensor Efficiency Parameter, BE%

This parameter (Equation (6)) incorporates influences from enzyme loading and substrate affinity. It is the most important of the different parameters studied here because it indicates how efficiently the biosensor enzyme composite layer converts Glu to electrochemical current (Equations (1)–(3)), and is the Glu linear region slope (Figure 1) normalized with respect to hydrogen peroxide sensitivity for each individual biosensor. This normalization is a key feature in biosensor optimization for LIVE applications because hydrogen peroxide is generated in living tissues from a variety of metabolic processes [70], and therefore may act as a biosensor interference species under some conditions. Enhancing the biosensor signal, therefore, using a strategy which also increases the hydrogen peroxide current, such as roughening the electrode surface [71], would not be as effective as augmenting the efficiency of the enzyme layer in converting Glu to hydrogen peroxide, which was the main goal of the present work.

In line with previous results for wild-type GluOx [47], and enzyme loading and affinity results above for recombinant GluOx, the biosensor design, which gave the highest value of biosensor efficiency (BE%), was Pt_C_/PEI_2_/GluOx_5_ (45% ± 3%, *n* = 4), which halved to 22% ± 4% (*n* = 4) by day 10, and reflecting mainly loss of active enzyme from the biosensor surface (see Figure 2b). This contrasts with the least stable PEI-containing design (Pt_C_/PEI_5_/GluOx_5_) where the maximum BE% was 35% ± 1% (*n* = 3), which halved by day 3. On the basis of these results, it was decided to take the PEI_2_/GluOx_5_ design forward, unless otherwise stated, for further optimization to increase enzyme loading, efficiency, and biosensor stability.

### 3.2. Biosensor Performance in the Presence and Absence of the Permselective PoPD Layer

Because an interference-blocking layer is necessary in biosensors for LIVE applications, the effects of electrosynthesized PoPD (a well-established permselective polymer for neurochemical monitoring *in vivo* [44,72,73,74,75,76]) on the biosensor enzyme performance was investigated. The electropolymerization of PoPD, and its subsequent conditioning, is time consuming (3 h). Thus, it was hoped that the more facile PoPD-free biosensor designs could be optimized, and then PoPD introduced into the final design. In addition, the exclusion of PoPD in the design could be further justified as the interference-rejection properties of the Glu biosensor was not the main focus here, which was the effects of a range of crosslinkers on the Glu biosensor sensitivity and stability. The performance of various Pt_C_/PEI_n_/GluOx_m_ designs was therefore tested in the presence and absence of PoPD to investigate whether the polymer was having a significant impact on the key enzyme-related biosensor parameter, BE%.

An unpaired *t*-test analysis showed that PoPD had only marginal beneficial effects on both Enz_act_ (~20% increase) and *K*_M_ (~20% decrease). However, BE%, which is a combination of these two parameters (Equation (6)), displayed a significant enhancement (64% ± 6%, *n* = 27; *p* < 0.001) when PoPD was incorporated into a variety of PEI/GluOx biosensor designs, indicating that the inclusion of PoPD is beneficial by improving the overall efficiency of the biosensor enzyme layer. Therefore, PoPD was included in the previously chosen design (giving Pt_C_/PoPD/PEI_2_/GluOx_5_) for all further stages of biosensor optimization.

### 3.3. Within-Study Comparison of Native and Recombinant GluOx Forms

Many previously reported descriptions of PEI-containing Glu biosensors were based on wild-type GluOx [26,49,77,78]. Since recombinant enzyme is now more readily available [53], a comparison of biosensor parameters for native vs. recombinant GluOx was undertaken. To include the key property of stability, the range of analytical parameters were therefore reviewed following 11 days of successive calibrations for the partially optimized Pt_C_/PoPD/PEI_2_/GluOx_5_ design, containing either native or recombinant GluOx.

On day 11, the Enz_act_ value for the biosensor featuring recombinant GluOx (18 ± 3 mM, *n* = 4) was not statistically different from that containing the native form of GluOx (13 ± 5 mM, *n* = 3, *p* > 0.80). The *K*_M_ values on day 11 for the recombinant and native GluOx biosensor designs were also not statistically different from each other: 0.50 ± 0.05 mM (*n* = 4) and 0.44 ± 0.02 mM (*n* = 3), respectively (*p* > 0.26). Given the similarity of the loading and affinity characteristics of biosensors fabricated from these two GluOx forms, it was not surprising that the BE% values on day 11 for the recombinant and native GluOx biosensor designs were statistically the same: 26% ± 2% (*n* = 4) and 23% ± 8% (*n* = 3), respectively (*p* > 0.74).

It is clear, therefore, that the quality of the biosensor was not compromised by the use of recombinant GluOx. Because recombinant forms of GluOx are now widely available, and used by many laboratories [55,79,80], it was utilized in the further development of Glu biosensors described below. Specifically, a variety of crosslinking agents were tested in attempts to increase initial active enzyme loading, as well as mitigate its subsequent loss from the biosensor surface (see Figure 1 and Figure 2).

### 3.4. Glutaraldehyde

Glutaraldehyde (GA) is a 5-carbon dicarbonyl, which has been employed successfully in biosensor enzyme crosslinking in the past [81,82,83], and was used here in initial attempts to retain GluOx on the biosensor surface. Unfortunately, there are drawbacks associated with its use, such as the health hazards related to its handling and the unwanted partial deactivation of enzymes which may occur following the crosslinking reaction. For example, there was an increase in *K*_M_ when the chosen biosensor design (Pt_C_/PoPD/PEI_2_/GluOx_5_) was exposed to 1% GA for 5 s and left to dry for 10 min: from 0.4 ± 0.1 mM to 1.3 ± 0.2 mM (*p* < 0.003, *n* = 6).

In deference to the longstanding use of GA in biosensor design [82], an attempt was made to exploit this agent and minimize the amount of GluOx damage by controlling the GA reaction. In contrast to previous studies where very low concentrations of GA have been used, together with incorporation of a non-enzyme protein (usually bovine serum albumin) into the crosslinking medium to limit enzyme deactivation [84,85], GluOx protection was attempted here by adjusting the exposure time of the biosensor to GA and by controlling the subsequent crosslinking reaction by quenching in water for predetermined times. Therefore, GA was introduced to the Pt_C_/PoPD/PEI_2_/GluOx_5_ design to establish which combination of exposure and quenching times would result in the most favorable outcome.

Initially, biosensors were exposed to a concentration of 25% GA for either 5 or 10 s, followed by quenching times of 10 min or 1 h. The initial focus was the *K*_M_ values as they represent the change in substrate affinity for the GluOx. The *K*_M_ for biosensors exposed to GA for 5 s and quenched for 1 h was 0.50 ± 0.06 mM (*n* = 8), whereas a quenching time of 10 min led to the indistinguishably similar *K*_M_ value: 0.58 ± 0.16 mM (*n* = 8, *p* > 0.6). These results indicate that 10 min was sufficient for the quenching of the crosslinking reaction to a level that protected the GluOx, and this quenching time was used for subsequent GA experiments. A longer exposure time of 10 s, followed by quenching, resulted in a *K*_M_ of 0.69 ± 0.06 mM (*n* = 8, *p* < 0.05), indicating a marginally significant loss of enzyme affinity for its substrate and an upper limit to exposure time.

A lower GA concentration (1% GA) was also examined and compared with both 25% GA and GA-free biosensor designs. The effects of varying the GA concentrations (0%, 1% and 25%) on the enzyme-related biosensor parameters are described in more detail in the following section.

#### Effect of Glutaraldehyde on BE%

As illustrated in Section 3.2, the biosensor efficiency parameter BE% (Equation (6)) is quite a sensitive index of the effects of biosensor modification on the response of the enzyme composite layer to substrate, and was therefore analyzed in detail here to probe the influence of GA (see Table 1).

The BE% value of the crosslinker-free design (Pt_C_/PoPD/PEI_2_/GluOx_5_) decreased from 41% ± 2% (*n* = 16) on day 0 to 26% ± 3% (*n* = 14) on day 10, representing a 36% loss of HP-normalized sensitivity over the 10 day period (*p* < 0.001; see Table 1). The 1% GA treatment did not affect BE% on day 0 compared with the GA-free design (*p* > 0.61); however, neither did it affect the loss of efficiency over the 10 days (38% loss, *p* < 0.03). Again, the 25% GA treatment did not affect BE% on day 0 compared with the GA-free design (*p* > 0.57), highlighting the protective effect of the quenching protocol. However, in contrast to the 1% GA treatment, exposure of the biosensor to this high concentration of GA completely prevented the decrease in BE% over the 10 days (Table 1).

### 3.5. Effect of Other Crosslinking Agents on BE%

The use of GA in the biosensor design was deemed to be successful because it led to a more stable biosensor when compared to the corresponding crosslinker-free design (Table 1). The large increase in *K*_M_ observed under some crosslinking conditions was alleviated by controlling the exposure time to GA and by quenching the reaction. However, due to the hazards associated with the use of GA, other crosslinkers were investigated, starting with polyethylene glycol, PEG.

#### 3.5.1. Polyethylene Glycol, PEG

PEG, which is capable of crosslinking to GluOx through its reaction with carboxyl groups present on GluOx, was an attractive modifier here as it is nontoxic and has been shown not to affect the biological activities of enzymes [86]. Both the absolute values and the time course for changes in BE% were statistically the same when either 0.1% or 1% PEG was used as the crosslinker (Table 1). As observed for biosensors incorporating GA, day 0 BE% values were indistinguishable from the crosslinker-free design. However, the PEG-based designs showed no ability to protect the biosensor enzyme layer from loss of sensitivity over the initial 10-day period.

#### 3.5.2. Poly(ethyleneglycol) Diglycidyl Ether, PEGDE

Activated PEG in the form of PEGDE is a highly soluble polymer and is capable of crosslinking with enzymes through covalent and non-covalent interactions. PEGDE is reactive towards the various functional groups on proteins (amines, carboxyl and hydroxyl groups) owing to the highly reactive epoxy terminal groups present on PEGDE. This crosslinking agent has been used successfully in the past with glucose oxidase [87], and more recently by Vasylieva and co-workers with a variety of oxidase enzymes [88]. PEGDE was used here in two ways: at room temperature; and at elevated temperatures whereby the sensors were cured for 2 h at 55 °C to allow for the possibility of a more effective crosslinking reaction [88].

There was no statistically significant difference between either the two PEGDE concentrations (0.1% or 1%) or crosslinking temperature on day 0 when compared with the crosslinker-free design (see Table 1). In contrast to PEG designs, all PEGDE configurations were able to prevent any loss of Glu sensitivity over the first 10 days of operation, with 1% PEGDE being marginally more effective than the 0.1% crosslinking solution. The data in Table 1 show clearly that PEGDE was as effective in stabilizing the biosensors as quenched GA crosslinking, without the corresponding handling hazards.

### 3.6. Limit of Detection, and Response Time

There was no significant difference between the LOD values (μM Glu) determined for sample sets of each key design with the generic form Pt_C_/PoPD/PEI_2_/GluOx_5_/XL, where XL is the crosslinking agent: no crosslinking agent (0.42 ± 0.08, *n* = 13); GA (0.22 ± 0.04, *n* = 4); PEG (0.16 ± 0.03, *n* = 6); PEGDE (0.17 ± 0.05, *n* = 6). These values indicate that the sensitivity is adequate for LIVE applications in brain ECF, where baseline Glu levels have been estimated to be in the low micromolar range (see Section 1). This is a necessary, but not sufficient, criterion for successful LIVE monitoring, where fast response time and long-term stability are also critical requirements.

The time response (*t*_90%_) of the crosslinker-free design (Pt_C_/PoPD/PEI_2_/GluOx_5_) was fast: 0.9 ± 0.2 s, *n* = 6, which is consistent with previous findings for other configurations of Glu biosensors based on wild-type GluOx and the ultrathin PoPD permselective polymer [50]. Crosslinking the PEI-GluOx layer with GA had a non-significant effect on *t*_90%_ values (1.2 ± 0.1 s, *n* = 4; *p* > 0.28). The effect of the two milder crosslinking agents (PEG and PEGDE) were similar, and were therefore pooled: 0.8 ± 0.1 s, *n* = 27. This was statistically significantly better than GA (*p* < 0.005), and the same as the crosslinker-free configuration (*p* > 0.67). This sub-second response time for the chosen crosslinker PEGDE is adequate for monitoring Glu concentration dynamics in future studies of brain extracellular fluid where diffusion from the synapse [89] dampens the sub-millisecond transients observed within the synaptic cleft [90]. These response-time results also revealed an additional drawback of using GA as the crosslinking agent, highlighting the advantages of PEDGE.

### 3.7. Stability of Selected Biosensor Designs up to Day 90

The data in Figure 1 and Figure 2, and Table 1, show that even biosensors incorporating the enzyme stability agent, PEI, lost a large fraction of GluOx activity from the electrode surface over a 10-day period of calibration when no crosslinker was used in their fabrication. The inclusion of a variety of crosslinking agents (Table 1) prevented loss of Glu sensitivity for at least 10 days, which is an adequate window of opportunity for a range neurochemical monitoring studies *in vivo*. To investigate the stability further, however, calibrations were performed on day 90 (following storage at 4 °C) for a selection of these biosensor designs, including heat-treated and non heat-treated PEGDE, and PEG-containing biosensors.

There was a general tendency for *K*_M_ to increase by day 90. There was no clear trend between the different crosslinkers, and the values were therefore pooled: 0.8 ± 0.1 mM (*n* = 25). Compared with the crosslinker-free configuration on day 0 (see Section 3.1.1), this day 90 average represented only a marginally significant doubling (*p* < 0.06) of *K*_M_ over 3 months of repeated calibrations and storage, and is a testament to the intrinsic stability of this surface-immobilized recombinant enzyme.

Throughout this biosensor development study, BE% was used to highlight the contribution of the enzyme activity to the biosensor signal by normalizing the LRS response with respect to HP sensitivity. In future neurochemical applications, however, the time course of HP sensitivity for implanted biosensors cannot be determined after implantation. Only the raw output of the biosensor will be available in attempts to monitor the concentration dynamics of Glu in brain extracellular fluid. For this reason, the Glu LRS for a range of crosslinked biosensors was used to estimate their stability on day 90, and compared with the day 10 responses discussed above. There was no statistically significant change in Glu LRS sensitivity on day 90 for the two benign agents crosslinked at room temperature: PEG (from 56 ± 9 to 49 ± 9 mA·cm^−2^·M^−1^, *n* = 6; *p* > 0.63) and PEGDE (from 51 ± 4 to 57 ± 6 mA·cm^−2^·M^−1^, *n* = 8; *p* > 0.39). In contrast, for biosensors with PEGDE crosslinked at 55 °C, the LRS value halved over the same period.

In addition to changes in Glu sensitivity, the time response was also investigated over this period. For the crosslinker of choice (PEGDE, *n* = 10) the *t*_90%_ values were: 0.6 ± 0.1 s, 0.9 ± 0.2 s, and 1.8 ± 0.4 s on days 0, 10 and 90, respectively. Thus, the Pt_C_/PoPD/PEI_2_/GluOx_5_/PEGDE glutamate biosensor design displayed sub-second time responses up to day 10, and although the day 90 value was significantly greater than that for day 0 (*p* < 0.01), a value <2 s is adequate for future LIVE applications.

## 4. Conclusions

Glutamate biosensors fabricated using both wild type and a recombinant form of GluOx were compared in terms of enzyme loading, substrate affinity and biosensor efficiency parameters, and found to be statistically equivalent. This latter, more readily available, source of GluOx can therefore be used in biosensor development without compromising analytical performance.

Comparison of a range of biosensor fabrication components showed that both the polycationic stabilizer PEI and the permselective electrosynthesized polymer PoPD enhanced the efficiency of the recombinant GluOx-based biosensor, as measured by its ability to convert the target analyte into current. Optimization of the different components yielded a biosensor of the form, Pt_C_/PoPD/PEI_2_/GluOx_5_ which, however, lost ~50% of its active surface enzyme over a 10-day period. The classic crosslinking agent, GA, was applied to this biosensor design using a quenching protocol that protected the substrate affinity parameter, *K*_M_, and maintained the activity of surface GluOx. However, there were two drawbacks to GA crosslinking: the biosensor time response was slowed, and there are hazards associated with handling GA. As alternatives, therefore, both unactivated and activated PEG (PEGDE) were tested as crosslinking agents, and using a range of criteria, PEGDE was found to be superior. The resulting biosensor design (Pt_C_/PoPD/PEI_2_/GluOx_5_/PEGDE) displayed good sensitivity (LOD < 0.2 μM), response time (*t*_90%_ < 1 s) and stability over a 90-day period, making it an attractive candidate for future long-term *in vivo* electrochemical monitoring of Glu concentration dynamics in brain extracellular fluid.

## Figures and Tables

**Table 1:** BE% (Equation (6)) for the biosensor designs Pt_C_/PoPD/PEI_2_/GluOx_5_/XL fabricated using a range of crosslinkers (XLs) at different concentrations and crosslinking conditions determined on day 0 and day 10. ΔBE% (day 0) is the difference in the day-0 BE% value compared with the crosslinker-free design. The 10-day stability value was calculated as the change in BE% on day 10 compared with that of day 0 for each biosensor design. The results for PEGDE crosslinked at room temperature or at 55 °C were statistically the same, and were, therefore, pooled.

Crosslinker	BE% (Day 0)	BE% (Day 10)	ΔBE% (Day 0)	*p*-Value	10-Day Stability	*p*-Value
none	41 ± 2 (*n* = 16)	26 ± 3 (*n* = 14)	0	N/A	−15 ± 4	<0.001
GA (1%)	37 ± 1 (*n* = 2)	23 ± 2 (*n* = 2)	−4 ± 2	>0.61	−14 ± 3	<0.03
GA (25%)	39 ± 3 (*n* = 8)	45 ± 3 (*n* = 6)	−3 ± 4	>0.57	+6 ± 4	>0.19
PEG (0.1%)	45 ± 2 (*n* = 3)	19 ± 6 (*n* = 3)	+4 ± 3	>0.41	−26 ± 5	<0.02
PEG (1%)	45 ± 8 (*n* = 3)	21 ± 6 (*n* = 3)	+4 ± 7	>0.46	−24 ± 9	<0.07
PEGDE (0.1%)	39 ± 2 (*n* = 7)	34 ± 3 (*n* = 7)	−2 ± 3	>0.55	−5 ± 4	>0.28
PEGDE (1%)	34 ± 2 (*n* = 12)	35 ± 3 (*n* = 12)	−7 ± 3	<0.05	+1 ± 4	>0.78
